# Overuse or underuse? Use of healthcare services among irregular migrants in a north-eastern Spanish region

**DOI:** 10.1186/s12939-020-01373-3

**Published:** 2021-01-20

**Authors:** Luis Andrés Gimeno-Feliu, Marta Pastor-Sanz, Beatriz Poblador-Plou, Amaia Calderón-Larrañaga, Esperanza Díaz, Alexandra Prados-Torres

**Affiliations:** 1 EpiChron Research Group on Chronic Diseases, Aragón Health Sciences Institute (IACS), IIS Aragón, Miguel Servet University Hospital, Zaragoza, Spain; 2 Aragón Healthcare Service, San Pablo Health Centre, Zaragoza, Spain; 3 Health Services Research on Chronic Patients Network (REDISSEC), Carlos III Health Institute, Madrid, Spain; 4 Department of Medicine, Psychiatry and Dermatology, University of Zaragoza, Zaragoza, Spain; 5 Aragón Healthcare Service, Utrillas Health Centre, Zaragoza, Spain; 6 Aging Research Center, Department of Neurobiology, Care Sciences and Society, Karolinska Institutet & Stockholm University, Stockholm, Sweden; 7 Research Group for General Practice, Department of Global Public Health and Primary Care, University of Bergen, Bergen, Norway; 8 Norwegian Centre for Minority Health Research, Oslo, Norway

**Keywords:** Emigration and immigration, Undocumented migrant, Healthcare disparities, Health services accessibility, Hospitalization, Primary healthcare, Emergency service, Drug utilization, Healthcare use, Spain

## Abstract

**Background:**

There is little verified information on global healthcare utilization by irregular migrants. Understanding how immigrants use healthcare services based on their needs is crucial to establish effective health policy. We compared healthcare utilization between irregular migrants, documented migrants, and Spanish nationals in a Spanish autonomous community.

**Methods:**

This retrospective, observational study included the total adult population of Aragon, Spain: 930,131 Spanish nationals; 123,432 documented migrants; and 17,152 irregular migrants. Healthcare utilization data were compared between irregular migrants, documented migrants and Spanish nationals for the year 2011. Multivariable standard or zero-inflated negative binomial regression models were generated, adjusting for age, sex, length of stay, and morbidity burden.

**Results:**

The average annual use of healthcare services was lower for irregular migrants than for documented migrants and Spanish nationals at all levels of care analyzed: primary care (0.5 vs 4 vs 6.7 visits); specialized care (0.2 vs 1.8 vs 2.9 visits); planned hospital admissions (0.3 vs 2 vs 4.23 per 100 individuals), unplanned hospital admissions (0.5 vs 3.5 vs 5.2 per 100 individuals), and emergency room visits (0.4 vs 2.8 vs 2.8 per 10 individuals). The average annual prescription drug expenditure was also lower for irregular migrants (€9) than for documented migrants (€77) and Spanish nationals (€367). These differences were only partially attenuated after adjusting for age, sex, and morbidity burden.

**Conclusions:**

Under conditions of equal access, healthcare utilization is much lower among irregular migrants than Spanish nationals (and lower than that of documented migrants), regardless of country of origin or length of stay in Spain.

**Supplementary Information:**

The online version contains supplementary material available at 10.1186/s12939-020-01373-3.

## Background

According to the 2018 United Nations Migration Report, the number of migrants worldwide reached 244 million in 2015 and is expected to increase further [1]. A small but notable portion of the general migrant population consists of migrants without legal authorization to reside in their host country. The term *irregular migrant* refers to “a person who, owing to unauthorized entry, breach of a condition of entry, or the expiry of his or her visa, lacks legal status in a transit or host country” [2]*.* It includes persons who (a) lack the necessary documentation to legally enter a country but do so clandestinely; (b) enter or stay in a country using fraudulent documentation; or (c) after entering a country with valid legal documentation, stay beyond the period authorized or otherwise violate the terms of entry and remain without authorization [2]. This term is considered a synonym of “undocumented migrant”, which refers to the individual’s administrative situation and is one of the most widely used and accepted terms [2–4]. The term encompasses visa “overstayers”, those who have lost resident status, rejected asylum seekers, and individuals who have entered a country illegally [3–5]. In 2008 an estimated 1.9–3.8 million irregular migrants were living in the European Union [4].

**Fig. 1:**
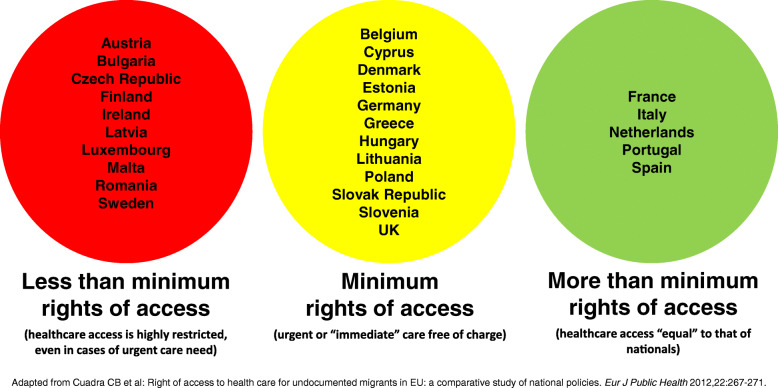
National policy regarding rights of access to health care for irregular migrants in the EU

In recent years, several countries have restricted public healthcare access of this population, arguing that migrants migrate to host countries to avail of treatment for pre-existing medical conditions [6–8]. Cuadra et al. classified EU countries into three main groups according to the availability of healthcare services to migrants [9] (summarized in Fig. 1). Immigrants in Spain account for 12.2% of the population (12.7% in Aragon), and migrate to Spain primarily for economic reasons [10]. The Spanish national health system provides universal coverage and is almost fully funded by taxes. Care provision is free of charge at the point of delivery, resulting in a practically free system. Primary care centres serve as gatekeepers and are distributed to ensure appropriate geographical coverage. While the Spanish public health system is decentralized and managed by the regional government of each autonomous community, the legislation governing access to the national health system applies to the entire state [11]. Between 2000 and 2012, immigrants were guaranteed legal access to the same healthcare services as Spanish nationals, regardless of legal status. During that period, Spain was one of the most progressive countries in the world in terms of healthcare access of irregular migrants [12]. In 2012, in response to the persistent effects of the 2008 economic crisis, a central government decree withdrew this right, invalidating the health cards of irregular migrants [13–15]. The government argued, without offering evidence, that this radical change in healthcare access would help limit alleged “health tourism” and improve the sustainability of Spain’s national health system. This measure was accompanied by a raft of other cutbacks affecting healthcare and social services. According to reports by the Spanish government, this policy affected 870,000 irregular migrants [13]. Since then, there has been continued political debate over the restoration of universal healthcare coverage.

As recommended by the WHO and other Human Rights organizations, healthcare provision is the minimum condition to guarantee equity in health [16]. In 2018, the socialist government of Pedro Sánchez passed Royal Decree Law 7/2018 on universal access to the national health system. While the resulting changes improved healthcare access, they did not restore universal health coverage across Spain. Royal Decree Law 7/2018 is currently being revised by the Spanish parliament, and has become a first-order political issue, given the widespread trend in Europe and beyond of withholding universal health coverage from irregular migrants [7, 17, 18].

Published data on the use of healthcare services by irregular migrants is scarce [5, 17]. A recent systematic review by Winters et al. highlighted the paucity of such studies [8]. Moreover, the findings of the few studies conducted to date are limited by small sample populations, a lack of adjustment for confounding factors, and an absence of control groups, as emphasized in a scoping review by Woodward et al. [19].

By tracing irregular migrants whose Spanish public health cards were invalidated in 2012, it is possible to examine healthcare use during the previous year, when there was no distinction between irregular migrants, documented migrants, and Spanish nationals in terms of public healthcare access. Our study analyses the real use of the Spanish public healthcare system by a large cohort of migrants (the largest published to date), including undocumented migrants, during a period (2011) when migrants had unlimited health system access regardless of legal status. Given that few national healthcare systems offer universal coverage to irregular migrants, this analysis provides an important window into healthcare usage in this specific context. The findings may therefore be of interest to policymakers and researchers seeking to improve healthcare systems and ensure health equity for the most disadvantaged populations.

## Methods

This cross-sectional population-based retrospective study analyzed clinical and administrative data from individuals assigned to all public primary care (PC) centres in Aragon, Spain, in 2011. These data were originally collected as part of the EpiChron Cohort, which gathers demographic, clinical, and pharmaceutical data from electronic health records (EHRs) and the health insurance database for almost all inhabitants of Aragon, using a unique anonymized personal identification code [20, 21]. The Aragon health service is part of the Spanish national health system, which provides free care and medical testing, and is funded by taxes. Care provision is free of charge at the point of delivery, resulting in a practically free system [22]. PC centres serve as gatekeepers and guarantee appropriate geographical coverage. Secondary care is provided through ambulatory specialized care, hospitals, and emergency rooms. Pharmaceuticals prescribed to those under 65 require a co-payment of 40% of the retail price (or less in the case of chronic medication); medication is otherwise free of charge at the point of delivery.

For each patient aged 18 and older, demographic variables including age, sex, country of birth, and length of residence in Aragon were extracted from the health insurance database for the year 2011. Immigrants were defined as any foreign-born person, regardless of nationality or duration of residence in Spain (2). The study population was categorized as Spanish nationals, documented migrants, or irregular migrants, defined as any individual whose health card was invalidated as a result of Royal Decree-Law 16/2012 [13]. The migrant population was classified according to their area of origin (Africa, Asia, Eastern Europe, Latin America, and Western Europe & North America).

Diagnostic data were extracted from EHRs and from the Hospital Minimum Basic Dataset (Spanish acronym, CMBD). In the former, diseases are registered according to the International Classification of Primary Care, Version 1 (ICPC-1). The latter gathers the diagnoses of patients discharged from all public and private hospitals, coded using the Clinical Modification-Ninth Revision of the International Classification of Diseases (ICD-9-CM). The Adjusted Clinical Group (ACG) System® was used to group all ICPC and ICD diagnostic codes based on duration, severity, diagnostic certainty, aetiology, and specialized care involvement. A unique ACG category was assigned to each individual based on age, sex, and all diagnoses registered during the study period. Individuals within a given ACG show similar patterns of morbidity and resource utilization over a given year. ACGs with similar expected use of resources were aggregated into one of the six so-called resource utilization bands (RUB 0 = non users; RUB 1 = healthy users; RUB 2 = low morbidity; RUB 3 = moderate morbidity; RUB 4 = high morbidity; and RUB 5 = very high morbidity). Each individual was thus additionally assigned a RUB category.

PC use was defined as the number of visits to the PC doctor and nurse, including on-demand, scheduled, emergency, and home visits. Specialized care utilization was measured as the total number of visits to any specialist. Hospital care included planned and unplanned admissions, and the total number of hospital days. The use of emergency room services was measured as the total number of visits and priority visits. Priority visits were identified based on the triage level established by the health service of Aragon; out of the five categories listed, levels 1–3 are assigned to priority visits. Prescription drug use was measured as the total annual expenditure using recommended retail drug prices [23].

The study was approved by the Clinical Research Ethics Committee of Aragon (CEICA).

### Statistical analysis

The use of each level of care and prescription drug expenditure were determined according to migrant status. Given the over-dispersion in the distribution of the outcome variables, negative binomial regression models were applied to determine the association between the latter and individuals' area of origin through incidence rate ratios (IRR) and 95% confidence intervals. Evaluation of the over-dispersion parameter alpha allowed verification of the adequacy of the negative binomial regression models with respect to Poisson count models. Because the observed outcome data often contained a higher relative frequency of zeros than is consistent with negative binomial model specifications, zero-inflated models were used. The Vuong closeness test was used to assess the appropriateness of the zero-inflated models. When the Vuong test was not significant, and produced large negative values, standard negative binomial models were employed. When non-concave regions repeatedly appeared, use of a stepping algorithm as an alternative to the standard maximum likelihood algorithm was permitted. Normal Poisson models and zero-inflated Poisson models were also performed (see Supplementary Table 2) as a sensitivity analysis. Moreover, we repeated our analysis of pharmacy costs using an ordinary least squares analysis (i.e. linear regression), the results of which are shown in Supplementary Table 4.

All models were stratified by sex and area of origin and adjusted for age, morbidity burden, and length of stay. Length of stay in Spain was categorized as < 5 or ≥5 years as previously described [24]. When analyzing subgroups based on sex and area of origin, the small sample size of some subgroups prevented convergence of the models. These results are therefore not presented (i.e. pharmacy use among Asian immigrants). All statistical analyses were performed using STATA / IC 12.

## Results

Data for 1,070,715 individuals were analyzed: 930,131 Spanish nationals; 123,432 documented migrants; and 17,152 irregular migrants. Table 1 presents the main demographic characteristics, morbidity burden, and healthcare utilization data for Spanish nationals and immigrants in Spain, without adjusting for any variable. The distribution of the migrant population according to area of origin is shown in Supplementary Table 1. The mean age of irregular and documented migrants was similar, and in both cases was lower than that of Spanish nationals. The percentage of women was lower among migrants than Spanish nationals, and was lowest among irregular migrants. Use of the public health system by irregular migrants was lower than that of documented migrants and Spanish nationals, in terms of both the number of visits and hospitalizations per year: PC, 0.5 vs 4.0 vs 6.7; outpatient hospital visits, 0.2 vs 1.8 vs 2.9; planned hospital admissions (per 100 individuals), 0.3 vs 2 vs 4.2; unplanned hospital admissions (per 100 individuals), 0.5 vs 3.5 vs 5.2; emergency room visits (per 10 individuals), 0.4 vs 2.8 vs 2.8. Pharmacy expenditure was also lower among irregular migrants than both documented migrants and Spanish nationals: €8.7 vs €77.4 vs €366.5. Length of stay (< 5 years vs ≥5 years) did not significantly influence the use of healthcare services by irregular migrants.

**Table 1:** Demographics, morbidity burden, and healthcare utilization for Spanish nationals and immigrants in Spain

	Spanish nationals	Foreign-born (all)		Africa		Asia		Eastern Europe		Latin America		Western Europe & North America	
		DM	IM	DM	IM	DM	IM	DM	IM	DM	IM	DM	IM
**Demographic information**													
**N**	930,131	123,432	17,152	29,643	2563	4621	327	39,678	7429	39,829	4643	9661	2190
**Women, %**	51.42	48.55	33.76	31.96	18.77	43.24	37.61	51.23	32.12	59.09	45.53	47.49	31.37
**Age**													
**Mean (SD)**	51.91 (0.020)	38.18 (0.034)	38.06 (0.083)	37.39 (0.063)	37.85 (0.204)	38.49 (0.184)	39.71 (0.664)	36.71 (0.053)	37.23 (0.118)	38.51 (0.063)	38.89 (0.170)	45. 12 (0.147)	39.16 (0.248)
**18–44 years, %**	39.71	74.11	76.10	78.27	80.53	72.80	69.11	78.79	78.58	71.39	72.73	53.97	70.68
**45–64 years, %**	32.14	22.94	22.11	19.48	17.17	23.31	26.91	20.26	20.62	25.14	24.6	35.26	26.94
**65+ years, %**	28.16	2.95	1.79	2.25	2.3	3.90	3.98	0.95	0.79	3.47	2.67	10.78	2.37
**Length of stay in Spain ≥5 years, %**	─	68.61	63.23	62.77	73.55	80.65	85.32	53.65	52.4	75.73	78.35	57.55	52.51
**Morbidity burden**													
**Healthy users/non-users, %**	21.15	36.02	75.29	36.64	73.16	47.72	79.51	41.15	79.26	29.92	68.49	32.61	78.13
**Low/moderate morbidity, %**	72.03	60.54	24.31	59.65	26.3	49.49	20.18	56.01	20.35	66.39	31.12	63.02	21.55
**High/very high morbidity, %**	6.81	3.44	0.4	3.71	0.55	2.79	0.31	2.84	0.39	3.68	0.39	4.38	0.32
**Use of Primary Care**													
**No visits to doctor, %**	22.83	34.19	86.5	33.35	79.17	46.09	90.83	38.32	87.66	29.18	85.89	34.71	91.78
**No visits to nurse, %**	48.55	71.42	95.56	70.8	93.64	80.09	97.86	74.69	95.76	69.3	95.48	64.47	96.94
**Mean (SD) # of visits to doctor, normal care**	6.7 (8.4)	4.0 (5.6)	0.5 (2.0)	4.1 (5.6)	0.8 (2.2)	2.7 (4.6)	0.2 (0.8)	3.3 (5.1)	0.5 (1.9)	4.5 (5.8)	0.6 (2.2)	4.5 (6.6)	0.3 (1.8)
**Mean (SD) # of visits to doctor, urgent care**	0.4 (1.0)	0.4 (1.1)	0.001 (0.1)	0.6 (1.3)	0.004 (0.1)	0.2 (0.9)	0 (0)	0.4 (1.0)	0.0003 (0.2)	0.3 (0.9)	0.0006 (0.04)	0.3 (1.0)	0.002 (0.1)
**Mean (SD) # of visits to nurse**	3.7 (7.7)	1.0 (3.5)	0.1 (0.7)	1.1 (3.5)	0.1 (0.8)	0.8 (3.4)	0.6 (5.1)	0.8 (3.3)	0.1 (0.8)	1.1 (3.3)	0.1 (0.6)	1.8 (4.8)	0.1 (0.7)
**Use of Specialized Care**													
**No visits, %**	46.86	61.15	93.48	66.52	92.51	69.88	97.25	65.37	94.18	52.33	91.19	59.57	96.48
**Mean (SD) # of visits**	2.9 (4.9)	1.8 (3.7)	0.2 (1.3)	1.5 (3.6)	0.3 (1.6)	1.4 (3.5)	0.1 (0.7)	1.4 (3.3)	0.2 (1.2)	2.2 (4.0)	0.3 (1.5)	2.1 (4.3)	0.1 (0.9)
**Use of Hospital Care**													
**Mean (SD) # of planned admissions/100 ind.**	4.2 (26.0)	2.0 (17.3)	0.3 (5.4)	1.5 (14.0)	0.3 (6.8)	1.4 (13.2)	0 (0)	1.8% (18.3)	0.2 (4.6)	2.6 (18.6)	0.4 (6.7)	2.8 (18.6)	0.1 (3.0)
**Mean (SD) # of unplanned admissions/100 ind.**	5.2 (28.4)	3.5 (21.1)	0.5 (8.1)	3.8 (21.8)	0.6 (7.9)	3.6 (21.5)	0.6 (7.8)	3.1 (19.8)	0.5 (8.4)	3.5 (21.1)	0.5 (7.5)	3.6 (24.1)	0.4 (8.8)
**Mean (SD) hospital stay, days**	6.7 (8.4)	4.7 (9.7)	6.0 (1.2)	4.4 (6.4)	8.5 (17.9)	5.0 (6.3)	4 (2.8)	4.6 (14.4)	4.6 (6.5)	4.5 (6.4)	6.5 (15.7)	6.1 (10.1)	6.7 (5.5)
**Use of Emergency Care**													
**No visits, %**	81.83	81.95	96.93	83.06	96.06	84.48	96.64	82.8	96.92	79.2	96.64	85.16	98.63
**Mean (SD) # of visits/10 ind.**	2.8 (8.0)	2.8 (7.8)	0.4 (3.2)	2.8 (8.2)	0.5 (3.3)	2.4 (7.1)	0.4 (2.2)	2.7 (7.5)	0.4 (3.4)	3.3 (8.0)	0.5 (3.2)	2.2 (6.9)	0.2 (2.5)
**High priority visits, %**	51.2	39.6	44.5	36.7	46.6	37.2	27.3	37.5	41.8	42.2	50.3	46.0	33.3
**Pharmacy expenditure**													
**Zero expenditure, %**	24.0	39.4	89.6	36.0	83.8	50.6	93.6	44.8	90.6	35.3	88.8	38.6	94.4
**Mean (SD) expenditure, €**	366.5 (763.4)	77.4 (302.4)	8.7 (90.1)	70.4 (314.3)	10.4 (70.0)	64.3 (248.2)	2.7 (16.0)	53.9 (225.8)	9.1 (115.2)	82.9 (279.9)	9.7 (73.8)	179.3 (543.0)	4.6 (34.9)

Abbreviations: *DM* documented migrant, *ind* individuals, *SD* standard deviation, *IM* irregular migrant

**Fig. 2:**
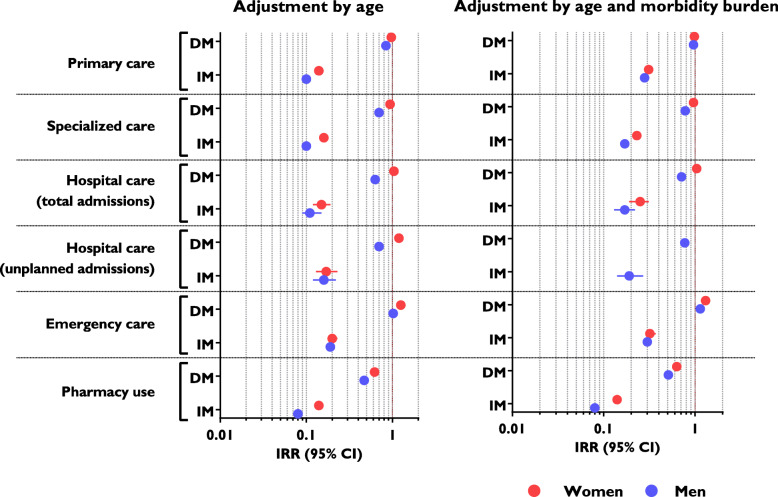
Use of healthcare services by immigrants according to legal status, and sex (expressed as incidence rate ratios, IRR): results of standard or zero-inflated negative binomial regression models. Abbreviations: DM, documented migrant; IM, irregular migrant. Data are normalized to corresponding values for Spanish nationals. In those cases where the Vuong test was statistically non-significant, standard negative binomial models were used

Figure 2 presents health system utilization data adjusted for age and stratified by sex, taking Spanish nationals as the reference population. The same data, adjusted for age and disease burden, are also presented. The age-adjusted models revealed lower use of healthcare services for irregular immigrants at all levels of care (IRR between 0.1–0.2 for both men and women). After adjusting for disease burden, these differences decreased, but remained significant, with IRR values between 0.15–0.3.

**Fig. 3:**
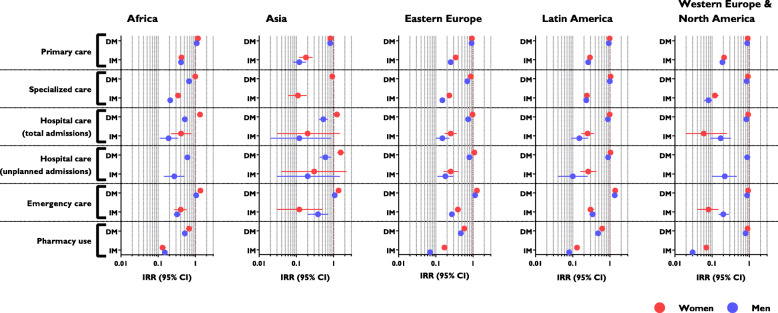
Use of healthcare services by immigrants according area of origin, legal status, and sex (expressed as incidence rate ratios, IRR): results of standard or zero-inflated negative binomial regression models. Abbreviations: DM, documented migrant; IM, irregular migrant. Models adjusted for age and morbidity burden. Data are normalized to corresponding values for Spanish nationals. In those cases where the Vuong test was statistically non-significant, standard negative binomial models were used

Figure 3 shows health system utilization adjusted for age and disease burden and stratified by area of origin and sex. In all cases, the use of healthcare services by irregular migrants was lower than that of Spanish nationals. Among irregular migrants, the use of healthcare services was highest among those from Africa, followed by Eastern Europe and Western Europe & North America. Healthcare use was lowest for those from Asia.

Supplementary Table 2 depicts the use of healthcare services by immigrants according to legal status (IRR), as determined using normal Poisson, zero-inflated Poisson, and standard or zero-inflated negative binomial regression models. The results obtained were very similar across models. Supplementary Table 3 depicts the use of healthcare services by immigrants according to legal status, adjusted for sex, age, morbidity burden, and additionally by area of origin (IRR). The results obtained were very similar to those shown in Fig. 1. The analysis of pharmacy costs was repeated using an ordinary least squares analysis (i.e. linear regression) (Supplementary Table 4), which revealed findings comparable to those obtained using the standard or zero-inflated negative binomial regression.

## Discussion

### Summary of the findings

#### Global use of healthcare services

The use of healthcare services by irregular migrants was very low, regardless of the level of care and after adjusting for age and disease burden (IRR between 0.15–0.3, after normalization to the corresponding values for Spanish nationals). The use of healthcare services among irregular migrants was also much lower than that observed for documented migrants. These results are consistent with the findings of a systematic review by Winters et al. [8]. Two questionnaire-based studies conducted in Portugal by Dias et al. reported findings similar to ours too, although the authors did not observe as great a difference between irregular and documented migrants [25, 26]. However, our results differ from those of Torres-Cantero et al., who interviewed 380 Ecuadorians in Madrid and observed no difference in healthcare utilization between irregular and documented migrants [27]. It is possible that in that study, limiting the study population to a very specific group that was interviewed on the street in a single district in Madrid may have led to some degree of selection or memory bias.

#### Primary care

PC utilization, after adjusting for disease burden, was much lower among irregular migrants than Spanish nationals (72% and 69% fewer visits for men and women, respectively). Recent studies performed in the Netherlands also reported reduced PC utilization among irregular migrants, although the differences with respect to documented migrants (37% [28] and 36% [29] fewer visits per year) were not as great as those observed in our population. It should be noted that, despite these marked differences, PC (together with emergency care) was the level of care for which the smallest proportional differences were observed between irregular migrants and Spanish nationals. This suggests greater equity of PC utilization in Spain, which has already been highlighted in other publications [30].

#### Hospitalizations, hospital visits, and emergency care

The numbers of total and unplanned hospitalizations and hospital outpatient visits were much lower among irregular migrants than either documented migrants or Spanish nationals. Although irregular migrants made fewer emergency room visits than either documented migrants or Spanish nationals, the differences observed for this level of care, as for PC, were relatively small. One potential explanation for this minimal difference is that emergency care is available 24 h a day, 7 days a week, and therefore irregular migrants with job insecurity can more easily avail of this type of care.

#### Pharmacy expenditure

Analysis of pharmacy expenditure revealed the greatest differences among groups of all outcomes analyzed. At the time at which this study was conducted, patients with a prescription from the Spanish national health service paid 40% of the cost of acute medication and 10% of the cost of chronic medication, with a maximum limit of €2.64 per package. Medication was free for inpatients and “exempt” groups (i.e. retirees and those who have disabilities or have had occupational accidents) [22]. The existence of this co-payment may have dissuaded irregular migrants from refilling their prescriptions.

### Interpretation of the findings

Several possible scenarios could help explain our findings. On the one hand, although the healthcare access of irregular migrants was equal to that of Spanish nationals and documented migrants, healthcare utilization by irregular migrants may be lower due to barriers to accessibility related to their work circumstances. For instance, those working in the underground economy are less likely to be able to avail of work leave [14, 31]. A qualitative study of irregular Latin American women working in Spain found that the main determinant of their lower healthcare utilization was precarious work and exploitation by employers [32]. In a study of irregular migrant women in the Netherlands, 40% reported barriers to healthcare access that included fear, shame, a lack of information, and financial difficulties [33].

Another potential barrier to healthcare access is the fear that the user’s data will be transferred to the authorities, potentially resulting in deportation from the country. Although such data sharing was prohibited in Spain at the time these data were gathered, the fact that this has occurred in other countries may have influenced irregular immigrants' way of thinking [8, 14, 31, 34]. Yet another potential barrier is difficulty communicating due to a lack of knowledge of the local language [31, 33, 35], although this effect would be expected to wane with increased length of stay in the host country, and this was not observed in our study population. Furthermore, in such case, a greater use of healthcare services would be expected in the Latin American population (who mostly speak Spanish), and this was not borne out by our findings (Fig. 2).

One other important factor is the emergence in various countries of “anti-immigration” policies, blaming immigrants, and especially irregular migrants, for numerous social and economic problems [31, 36]. These policies can reinforce feelings of rejection perceived by irregular migrants, making them more hesitant to fully exercise their rights, either out of fear or perceived “unworthiness” [14, 31, 36]. Larchanche et al. attribute this situation to the existence of “intangible obstacles”, such as stigmatization, structural violence, and fear [14]. Finally, “anti-immigration” rhetoric can influence health service personnel, giving rise to discriminatory and prejudiced attitudes that cause irregular migrants to stop availing of these services [14, 36].

In any case, the available evidence indicates that the lower use of healthcare services among irregular migrants is more closely linked to social factors related to their “illegal” status (e.g. a lack of worker’s rights, fear of deportation, economic difficulties, etc.) than to simply “being an immigrant”, which is likely to have a lesser impact [1, 8, 14, 31].

For both irregular and documented migrants, healthcare utilization was highest among those from Africa and lowest among those from Asia. The majority of Asians in our study population were from China, where there is a strong culture of the use of traditional Chinese medicine [37]. Furthermore, in Spain many immigrants of Asian origin work in small businesses with very long hours that may limit their access to healthcare services. The high relative use of healthcare services among immigrants of African origin is in line with the findings of a small Dutch study of female irregular migrants [33], and those of two studies that did not differentiate migrants according to legal status, one conducted in Spain by our group using administrative data [35] and another Portuguese questionnaire-based study [26]. We have no clear explanation for this particular finding. A study conducted in Portugal found that African migrants expressed greater satisfaction with the Portuguese healthcare system than those from Eastern Europe [25]. This difference warrants further specific research.

The lack of influence of length of stay on healthcare utilization among irregular migrants is notable. A previous study of this same cohort, in which there was no distinction between documented and irregular migrants, found that healthcare utilization increased with length of stay [35]. In line with our present findings, a Dutch study of 80 irregular migrant women (of mainly African and Eastern Europe origin) found that length of stay had no effect on the self-rated health status of irregular migrants, although the study in question lacked statistical power [34]. One possible reason is that documented migrants find it easier to integrate and assimilate to the cultural and societal norms of their host country than irregular migrants. This factor could contribute to the less medicalized health culture of irregular migrants as compared with the native population of their host country [23, 35]. Furthermore, documented migrants generally improve their economic situation over time, allowing them greater access to material goods such as medicines. This can lead to differences in expectations and priorities. It is also possible that documented migrants, who have a legal right to employment, may have access to less precarious jobs with corresponding worker’s rights, enabling them to take leave from work for medical consultations. Furthermore, as discussed above, irregular migrants may be more fearful of being detained or rejected by health officials and less aware about health related “deservingness” [14].

Analysis of sex-related differences revealed that the relative difference in healthcare utilization between migrants and Spanish nationals tended to be smaller for women than for men. This finding contradicts that of a Dutch study in which it was hypothesized that irregular migrant women have poorer access to healthcare services than their male counterparts [33].

### Strengths

These data, despite their retrospective nature, are particularly important as they constitute the largest series of healthcare data from undocumented migrants published to date [8], in a very specific context: a national healthcare system offering universal coverage. The results obtained from this analysis can therefore be of interest to policymakers and researchers seeking to improve universal healthcare systems and ensure health equity for disadvantaged populations based on the best available scientific evidence. These data, extracted from administrative databases, were previously collected as part of the EpiChron cohort [20]. Moreover, because the Spanish public health system provided migrants with universal coverage regardless of their legal status during the period to which these data correspond, the risk of selection bias in our dataset is limited. The dataset includes all individuals entitled to healthcare, regardless of whether they had any contact with the health system that year. The use of EHR data avoids biases associated with information provided directly by the patient.

Another key strength of our study is the comparison of healthcare utilization between irregular and documented migrants with a control group (i.e. Spanish nationals). The use of administrative data allowed us to study the impact of the length of stay. Moreover, thanks to the large sample size and diversity of the migrant population in Aragon, it was possible to further classify them by area of origin. By measuring individual-level morbidity burden using an internationally validated tool and data from EHRs, we ensured a broad and reliable assessment of the healthcare needs of the immigrant population [35]. Furthermore, by simultaneously analysing the use of primary care, specialized care, hospitalizations, emergency room visits, and prescription drug expenditure, our analysis allowed a global assessment of the Spanish health system. This global approach provides a comprehensive overview of the main health resources involved, allowing us to determine whether underuse of one resource can entail overuse of others [35, 38].

### Weaknesses

Several limitations of the present study should be noted. Our analyses did not consider socio-economic variables such as income or education level, inclusion of which could have helped identify some of the complex factors that condition the use of healthcare services [39, 40]. This important personal information is not recorded in Spanish healthcare databases and could not be obtained in any other way while preserving anonymity. The quality of healthcare data extracted from EHRs can also be called into question. However, the databases from which our data were acquired have been previously validated for use in comparative studies at the population level [38, 41]. It is possible that some irregular migrants may have left the region without notifying the public health system, and therefore their record may appear as active, despite the absence of any associated use of healthcare services. However, such cases are managed automatically by the health system of Aragon: health cards belonging to irregular migrants who do not renew their card every two years are cancelled automatically.

## Conclusions

Under conditions of equal access, the use of healthcare services by irregular migrants is markedly lower than that of Spanish nationals (as well as documented migrants), regardless of area of origin and length of stay in Spain. This lower healthcare utilization is likely related to the social consequences of their irregular legal status (e.g. job insecurity, economic difficulties, discrimination, fear of deportation), although the methodology used in our study does not allow for the identification of the specific underlying determinants. These findings are particularly relevant given the current political, socioeconomic, and healthcare challenges posed by a continual increase in the number of international migrants, and can help to facilitate evidence-based decision making by policymakers, both in Spain and worldwide, seeking to create systems that offer truly universal healthcare coverage that includes irregular migrants.

## Supplementary Information

**Additional file 1: Supplementary Table 1**: Distribution of the migrant population according to legal status and geographic area of origin**. Supplementary Table 2**: Use of healthcare services by immigrants according to legal status (incidence rate ratios, IRR). Results of standard Poisson, zero-inflated Poisson and standard or zero-inflated negative binomial models. **Supplementary Table 3**: Use of healthcare services by immigrants according legal status (incidence rate ratios, IRR). Results of standard or zero-inflated negative binomial regression models adjusted for sex, age, morbidity burden and, additionally, area of origin. **Supplementary Table 4**: Pharmacy use by immigrants according to legal status and sex. Results of linear regressions (expressed as β coefficients) and standard or zero-inflated negative binomial regression (expressed as incidence rate ratios, IRR) models.

## Data Availability

Data are available upon reasonable request.
