# Supplementary material for: Overuse or underuse? Use of healthcare services among irregular migrants in a north-eastern Spanish region
Source: Int J Equity Health. 2021 Jan 20;20:41. doi: 10.1186/s12939-020-01373-3 (PMC7816492; doi:10.1186/s12939-020-01373-3)
Supplement: Supplementary file 1 — Additional file 1: Supplementary Table 1: Distribution of the migrant population according to legal status and geographic area of origin. Supplementary Table 2: Use of healthcare services by immigrants according to legal status (incidence rate ratios, IRR). Results of standard Poisson, zero-inflated Poisson and standard or zero-inflated negative binomial models. Supplementary Table 3: Use of healthcare services by immigrants according legal status (incidence rate ratios, IRR). Results of standard or zero-inflated negative binomial regression models adjusted for sex, age, morbidity burden and, additionally, area of origin. Supplementary Table 4: Pharmacy use by immigrants according to legal status and sex. Results of linear regressions (expressed as β coefficients) and standard or zero-inflated negative binomial regression (expressed as incidence rate ratios, IRR) models. [file 12939_2020_1373_MOESM1_ESM.docx]

SUPPLEMENTARY TABLE 1: Distribution of the migrant population according to legal status and geographic area of origin.

|  | **DM** | **IM** | **Total** |
| --- | --- | --- | --- |
| **Africa** | **29.643** | **2.563** | **32.206** |
| Morocco | 14.683 | 977 | 15.660 |
| Algeria | 3.716 | 433 | 4.149 |
| Senegal | 2.749 | 223 | 2.972 |
| Gambia | 1.674 | 84 | 1.758 |
| Ghana | 1.502 | 145 | 1.647 |
| Mali | 1.192 | 91 | 1.283 |
| Equatorial Guinea | 935 | 151 | 1.086 |
| Guinea | 612 | 68 | 680 |
| Nigeria | 568 | 93 | 661 |
| Cape Verde | 419 | 33 | 452 |
| Mauritania | 378 | 42 | 420 |
| Cameroon | 178 | 19 | 197 |
| Egypt | 135 | 28 | 163 |
| Guinea-Bissau | 119 | 38 | 157 |
| Angola | 74 | 23 | 97 |
| Burkina Faso | 85 | 8 | 93 |
| Ivory Coast | 77 | 16 | 93 |
| Tunisia | 71 | 10 | 81 |
| Republic of the Congo | 63 | 12 | 75 |
| Western Sahara | 66 | 1 | 67 |
| Congo, Democratic Republic of | 41 | 7 | 48 |
| Niger | 35 | 4 | 39 |
| Sierra Leone | 19 | 20 | 39 |
| Liberia | 29 | 6 | 35 |
| Togo | 28 | 5 | 33 |
| South African Republic | 29 | 1 | 30 |
| Kenya | 25 | 5 | 30 |
| Mozambique | 16 | 5 | 21 |
| Gabon | 16 | 1 | 17 |
| Sudan | 14 | 0 | 14 |
| Mauritius | 11 | 0 | 11 |
| Benin | 10 | 1 | 11 |
| Central African Republic | 10 | 0 | 10 |
| Rwanda | 10 | 0 | 10 |
| Other countries | 54 | 13 | 67 |
| **Asia** | **4.621** | **327** | **4.948** |
| China | 2.971 | 203 | 3.174 |
| Pakistan | 753 | 30 | 783 |
| Syria | 127 | 6 | 133 |
| Armenia | 97 | 7 | 104 |
| India | 78 | 24 | 102 |
| Jordan | 80 | 5 | 85 |
| Lebanon | 64 | 1 | 65 |
| Philippines | 55 | 3 | 58 |
| Turkey | 49 | 7 | 56 |
| Bangladesh | 48 | 0 | 48 |
| Japan | 35 | 12 | 47 |
| Iran | 28 | 5 | 33 |
| Thailand | 21 | 3 | 24 |
| Laos | 21 | 0 | 21 |
| Israel | 19 | 2 | 21 |
| Korea, South | 19 | 0 | 19 |
| Korea, North | 15 | 4 | 19 |
| Kazakhstan | 14 | 3 | 17 |
| Uzbekistan | 14 | 2 | 16 |
| USSR | 10 | 4 | 14 |
| Indonesia | 9 | 1 | 10 |
| Other countries | 94 | 5 | 99 |
| **Eastern Europe** | **39.678** | **7.429** | **47.107** |
| Romania | 31.760 | 5.374 | 37.134 |
| Bulgaria | 3.010 | 451 | 3.461 |
| Poland | 1.714 | 1.089 | 2.803 |
| Ukraine | 1.644 | 201 | 1.845 |
| Russia | 581 | 69 | 650 |
| Moldova | 266 | 38 | 304 |
| Lithuania | 227 | 59 | 286 |
| Slovakia | 87 | 60 | 147 |
| Slovakia | 70 | 11 | 81 |
| Hungary | 61 | 27 | 88 |
| Albania | 51 | 12 | 63 |
| Bosnia and Herzegovina | 45 | 4 | 49 |
| Georgia | 43 | 11 | 54 |
| Latvia | 32 | 7 | 39 |
| Yugoslavia | 31 | 2 | 33 |
| Croatia | 17 | 6 | 23 |
| Serbia | 14 | 2 | 16 |
| Slovenia | 10 | 5 | 15 |
| Other countries | 15 | 1 | 16 |
| **Latin America** | **39.829** | **4.643** | **44.472** |
| Ecuador | 11.465 | 1.583 | 13.048 |
| Colombia | 7.823 | 1.017 | 8.840 |
| Dominican Republic | 2.968 | 134 | 3.102 |
| Argentina | 2.825 | 411 | 3.236 |
| Peru | 2.821 | 280 | 3.101 |
| Nicaragua | 2.501 | 132 | 2.633 |
| Brazil | 1.850 | 331 | 2.181 |
| Cuba | 1.787 | 123 | 1.910 |
| Venezuela | 1.262 | 116 | 1.378 |
| Bolivia | 950 | 121 | 1.071 |
| Chile | 916 | 119 | 1.035 |
| Uruguay | 688 | 61 | 749 |
| Honduras | 652 | 42 | 694 |
| Mexico | 467 | 111 | 578 |
| Paraguay | 295 | 39 | 334 |
| El Salvador | 227 | 6 | 233 |
| Guatemala | 91 | 4 | 95 |
| Panama | 84 | 5 | 89 |
| Costa Rica | 51 | 1 | 52 |
| Dominica | 48 | 4 | 52 |
| Puerto Rico | 42 | 1 | 43 |
| Other countries | 16 | 2 | 18 |
| **Western Europe & North America** | **9.661** | **2.190** | **11.851** |
| France | 3.302 | 252 | 3.554 |
| Portugal | 1.995 | 1.292 | 3.287 |
| Germany | 1.178 | 113 | 1.291 |
| Italy | 661 | 204 | 865 |
| Switzerland | 568 | 17 | 585 |
| United Kingdom | 561 | 97 | 658 |
| United States | 371 | 27 | 398 |
| Belgium | 250 | 31 | 281 |
| Netherlands | 239 | 45 | 284 |
| Czech Republic | 87 | 42 | 129 |
| Canada | 82 | 6 | 88 |
| Andorra | 65 | 2 | 67 |
| Australia | 61 | 1 | 62 |
| Ireland | 50 | 15 | 65 |
| Denmark | 38 | 3 | 41 |
| Sweden | 36 | 15 | 51 |
| Austria | 32 | 4 | 36 |
| Greece | 28 | 12 | 40 |
| Finland | 19 | 3 | 22 |
| Norway | 14 | 4 | 18 |
| Other countries | 24 | 5 | 29 |

Abbreviations: DM, documented migrant; IM, irregular migrant.

**SUPPLEMENTARY TABLE 2: Use of healthcare services by immigrants according to legal status (incidence rate ratios, IRR). Results of standard Poisson, zero-inflated Poisson and standard or zero-inflated negative binomial models.**

| **Outcome** | **DM** | | | **IM** | | |
| --- | --- | --- | --- | --- | --- | --- |
|  | **IRR** | **95% CI** | | **IRR** | **95% CI** | |
|  | **Standard Poisson models** | | | | | |
| **Primary care** | 0,975 | 0,973 | 0,978 | 0,319 | 0,313 | 0,326 |
| **Specialized care** | 0,903 | 0,899 | 0,907 | 0,226 | 0,219 | 0,234 |
| **Hospital care (total admissions)** | 0,938 | 0,915 | 0,963 | 0,187 | 0,157 | 0,222 |
| **Hospital care (unplanned admissions)** | 1,060 | 1,025 | 1,095 | 0,200 | 0,162 | 0,248 |
| **Emergency care** | 1,219 | 1,204 | 1,233 | 0,323 | 0,301 | 0,346 |
| **Pharmacy use** | 0,572 | 0,572 | 0,572 | 0,114 | 0,114 | 0,115 |
|  | **Zero-inflated Poisson models** | | | | | |
| **Primary care** | 0,966 | 0,963 | 0,969 | 0,387 | 0,377 | 0,396 |
| **Specialized care** | 0,957 | 0,952 | 0,962 | 0,148 | 0,142 | 0,153 |
| **Hospital care (total admissions)** | 0,922 | 0,897 | 0,947 | 0,188 | 0,158 | 0,224 |
| **Hospital care (unplanned admissions)** | 1,035 | 1,000 | 1,072 | 0,205 | 0,166 | 0,254 |
| **Emergency care** | 1,208 | 1,191 | 1,224 | 0,313 | 0,291 | 0,337 |
| **Pharmacy use** | 0,607 | 0,606 | 0,607 | 0,459 | 0,456 | 0,461 |
|  | **Standard or zero-inflated negative binomial models*** | | | | | |
| **Primary care** | 0,973 | 0,967 | 0,980 | 0,292 | 0,284 | 0,301 |
| **Specialized care** | 0,883 | 0,873 | 0,892 | 0,194 | 0,186 | 0,202 |
| **Hospital care (total admissions)** | 0,922 | 0,895 | 0,949 | 0,191 | 0,160 | 0,228 |
| **Hospital care (unplanned admissions)** | -- | -- | -- | -- | -- | -- |
| **Emergency care** | 1,225 | 1,206 | 1,244 | 0,309 | 0,286 | 0,333 |
| **Pharmacy use** | 0,572 | 0,566 | 0,579 | 0,099 | 0,096 | 0,102 |

Abbreviations: DM, documented migrant; IM, irregular migrant.

Models adjusted by sex, age and morbidity burden

*In those cases where the Vuong test was statistically non-significant showing large negative values, standard negative binomial models were used.

**SUPPLEMENTARY TABLE 3: Use of healthcare services by immigrants according legal status (incidence rate ratios, IRR). Results of standard or zero-inflated negative binomial regression models adjusted for sex, age, morbidity burden and, additionally, area of origin.**

| **Outcome** | **DM** | | | **IM** | | |
| --- | --- | --- | --- | --- | --- | --- |
|  | IRR | **95% CI** | | **IRR** | **95% CI** | |
| **Primary care** | 0,887 | 0,868 | 0,905 | 0,269 | 0,260 | 0,279 |
| **Specialized care** | 0,846 | 0,819 | 0,874 | 0,187 | 0,178 | 0,197 |
| **Hospital care (total admissions)** | 0,877 | 0,800 | 0,961 | 0,183 | 0,150 | 0,222 |
| **Hospital care (unplanned admissions)** | -- | -- | -- | -- | -- | -- |
| **Emergency care** | 0,891 | 0,844 | 0,941 | 0,228 | 0,208 | 0,249 |
| **Pharmacy use** | 0,744 | 0,719 | 0,770 | 0,132 | 0,126 | 0,138 |

Abbreviations: DM, documented migrant; IM, irregular migrant.

In those cases where the Vuong test was statistically non-significant showing large negative values, standard negative binomial models were used.

**SUPPLEMENTARY TABLE 4: Pharmacy use by immigrants according to legal status and sex. Results of linear regression (expressed as β coefficients) and standard or zero-inflated negative binomial regression (expressed as incidence rate ratios, IRR) models.**

|  | **MEN** | | | | | |
| --- | --- | --- | --- | --- | --- | --- |
|  | **DM** | | | **IM** | | |
|  | **β / IRR** | **95% CI** | | **β / IRR** | **95% CI** | |
| **Linear regression** (*adjusted for age*) | -79,603 | -84,925 | -74,281 | -127,194 | -138,874 | -115,515 |
| **Standard or zero-inflated negative binomial regression** (*adjusted for age*)* | 0,474 | 0,466 | 0,482 | 0,076 | 0,072 | 0,080 |
| **Linear regression** (*adjusted for age + morbidity burden*) | -51,591 | -56,708 | -46,473 | -40,344 | -51,725 | -28,963 |
| **Standard or zero-inflated negative binomial regression** (*adjusted for age + morbidity burden*)* | 0,511 | 0,503 | 0,520 | 0,079 | 0,076 | 0,082 |
|  | **WOMEN** | | | | | |
|  | **DM** | | | **IM** | | |
|  | **β / IRR** | **95% CI** | | **β / IRR** | **95% CI** | |
| **Linear regression** (*adjusted for age*) | -76,830 | -82,801 | -70,859 | -146,579 | -164,313 | -128,845 |
| **Standard or zero-inflated negative binomial regression** (*adjusted for age*)* | 0,615 | 0,607 | 0,624 | 0,136 | 0,128 | 0,144 |
| **Linear regression** (*adjusted for age + morbidity burden*) | -68,683 | -74,505 | -62,861 | -43,603 | -61,060 | -26,145 |
| **Standard or zero-inflated negative binomial regression** (*adjusted for age + morbidity burden*)* | 0,631 | 0,623 | 0,640 | 0,135 | 0,129 | 0,142 |

Abbreviations: DM, documented migrant; IM, irregular migrant.

*In those cases where the Vuong test was statistically non-significant showing large negative values, standard negative binomial models were used.
